# Miscellanies

**Published:** 1835-04-01

**Authors:** 


### MISCELLANIES.

### Birmingham School of Medicine.

We have perused, with much pleasure, an eloquent oration, delivered on the 6th of October last year, by Dr. John Johnstone, on the occasion of passing the laws and regulations of the Birmingham School of Medicine and Surgery. In this school, as in many other places, "narrow indeed were the means, few were the powers, and small the accommodations of those persons whose intentions are realized this day."—Though Birmingham, the metropolis of the West, has hitherto brought forth no Linacre to frame a royal college—no Harvey to embellish it with an immortal discovery—yet its founders may congratulate themselves on having laid the foundation of a temple of science that will radiate benefits on countless generations yet to come. It was only in 1828 that the Birmingham school originated. "In it there was neither monopoly, nor the appearance of monopoly. The lectureships were offered to *all* the physicians and surgeons of the hospital and dispensary, according to seniority." On this point we cannot agree with the talented orator. It does not fall to the lot of every individual to belong to an hospital or a dispensary—neither is it the inevitable consequence that talent, learning, and experience shall be exclusively concentrated in the said medical officers. But let that pass. Up to 1829, the School had only the convenience of "ONE ROOM FOR ALL ITS PURPOSES." The lecturer on anatomy offered to build a set of rooms, provided the body of lecturers guaranteed a certain rental; but this proposal appears to have failed, and aid was sought from the "neighbouring patrons of science." Succour was obtained from this source, and the institution attained its present form and features. Medals and prizes were offered and awarded by liberal individuals, and to these honorary stimuli the pupils have ably responded. The orator gratefully records the emphatic lecture on hernia delivered by Mr. Bransby Cooper, at the first meeting of the institution, and the admirable effects which it produced on the pupils. The fruits of the instruction communicated and received at this school appear to be of the most gratifying kind. Gentlemen have been there educated, who have been marked with pointed sanction and praise by the London College of Surgeons, and the Hall. And the neighbouring counties can boast of practitioners of the very first respectability and talent, emanating from the same ALMA MATER.

It appears that the greatest praise is due to Mr. Laws Cox, under whose fostering care a princely museum has sprung up, as it were, by magic. Stores of most curious and useful anatomical preparations have been accumulated—unrivalled wax models procured—a museum of natural history added—and a library to crown the whole.

The laws which are appended to this address appear to be devised by wisdom, and adapted to general utility. To this code of laws—or rather to the institution itself, we shall only add the sincere valediction—*esto perpetua!*

---

### POPULATION RETURNS.

From these voluminous reports, collected and arranged by Mr. Rickman, we shall here extract a few particulars relative to the comparative mortality in different parts of England. A wonderful influence is exerted on the health, habits, comforts, &c. of people by the coacervation or domiciliary isolation of families. Thus in England and Wales there are 117 families to 100 houses—in Scotland, 133 to 100—in Ireland, 110 to 100. But the circumstances in the three kingdoms are so very different and diverse, that no inference can be drawn from a comparison on this point. London and Liverpool, however, may be compared in this respect, as they are somewhat similarly situated. In London there are 171 families to 100 houses—and the annual mortality was 1 in 44 in the year 1830. In Liverpool there are only 131 families to 100 houses, and the mortality was 1 in 52 of the population during the same year. Hull has 134 families to 100 houses—and the mortality is 1 in 49. Bristol shews 131 families to 100 houses—the mortality 1 in 61. This shews that the degree of isolation will not account entirely for the degree of salubrity. Liverpool and Bristol are situated alike in this respect, and yet there is a great difference in the ratio of mortality. The late Dr. Currie has assigned one cause for the greater mortality of Liverpool—the residence of numerous families in cellars or under-ground apartments. Again, in Manchester there are 116 families to 100 houses, and the mortality is 1 in 30!* whilst in Birmingham, where there are 105 families to 100 houses, and the mortality is 1 in 68—not one half of the Manchester mortality! This enormous disproportion in our two great manufacturing towns, must be owing chiefly to the greater destruction of juvenile life in Manchester than Birmingham, for obvious reasons—namely the facility of employing young people in the former locality, and the intractability of the Birmingham material of manufacture, requiring adult hands. In the woollen manufactures the applicability of infant labour holds a middle-place, and the crowding of population and mortality are proportionately less. In LEEDS there are 111 families to 100 houses—and the mortality is 1 in 48.

What must be the influence of concentration, then, in Dublin, where 252 families are *compressed*, for they can hardly be said to live, in 100 houses? What in Edinburgh, where 310 families conglomerate in 100 houses? Or in Paisley, where the astonishing number of 360 families exist in 100 houses? We have not the means, at present, of estimating the actual mortality in these places. It is curious, and, at the same

---

* Is it not remarkable that Dr. Hawkins should have been so deceived about the mortality in Manchester, which he makes, if we recollect right, 1 in 79!

time, melancholy, to observe that, while in England and Wales the ratio of coacervation has been diminishing 2 per cent. in the ten years between the last two centuries, that ratio has been increasing in Scotland, at about 2 per cent. In the two kingdoms together, it is satisfactory to see that, during the last decenniad, whilst the proportion of houses building is very nearly the same, the uninhabited houses are decreasing 60 per cent.

The calculations which are and will be founded on these returns must operate a considerable revolution in the rates of insurance on lives, and the chances of survivorship. In earlier times the ratio of mortality was calculated chiefly from the returns of large cities, as most easily obtained; but now the case is quite altered, and indeed there can be no doubt that life itself has become more valuable—that is, of longer duration than it was half a century ago. It is evident that the public has, for very many years, been paying a much higher rate of insurance than was necessary or just—hence the general reduction of premiums which the competition of life-insurance-offices is now every where effecting.

---

### Introductory Lecture to a Course of Physiology and Pathology. By Dr. Graves.

It will not be expected that we should analyse an introductory lecture, which, like a King's speech on opening the Parliament, is usually composed of general expressions, from which little that is definite can be gleaned. The present lecture is one which is calculated to expand our ideas, and suggest a multitude of reflections. It is an attempt to afford a glimpse of the unbounded field which the medical philosopher has before him for his interminable investigations. The kingdom of animated nature is, of course, his country; and wherever he turns, he finds the subjects of his contemplation and investigation in swarms around him.

"In the warmer regions of the earth, where copious exhalations arise from rivers, swamps, and impenetrable forests, the insect inhabitants of the air swarm in such myriads, that they seem to the tortured traveller to fill the whole atmosphere, and at certain seasons of the year, their attacks become so troublesome and so incessant, that they force whole tribes of people to save themselves by a temporary migration.*

Even in the higher northern latitudes, the heat of the summer solstice calls to a short-lived existence swarms of mosquitoes, from which the Esquimaux, the Laplander, and the Samoied are forced to protect themselves by keeping their huts constantly filled with smoke. How far the upper regions of the atmosphere may be peopled has not been ascertained, but it seems improbable that winged insects exist to any great distance above the highest flight of the swallow and swift, although wanderers from the plains are occasionally met with at considerable heights on mountains. Thus, in traversing the highest accessible regions of the Alps and Pyrennees, I have met with flights of butterflies, the emblems of summer and of sunshine, migrating from some neighbouring valley, and, in their fluttering and devious course, almost touching with their fragile wings the hard surface of the glacier and never-melting snow, the types of cold and of winter.† De Luc, Ramond, Saussure, and Humboldt have all seen insects at heights equal to, or even exceeding, that of the summit of Mont Blanc. Of all animals the condor, or enormous vulture of the Andes, attains to the greatest elevation,

---

* " Humboldt relates this fact.

† " Ramond (*Voyages au Mont Perdu*, a work replete with original views and new facts) in climbing a very dangerous precipice, arrived at a spot where both advance and retreat seemed impossible. In this painful and hazardous situation, the appearance of a fly awakened a curious reflection. 'Durant cette inaction qui devenait d'autant plus penible qu'elle se prolongeait davantage, la mouche apiforme vint se poser auprès de moi et nettoyer ses petites ailes dont nous etions reduits à envier la puissance!' "

for Humboldt saw it describing with expanded wings its aërial circles far above the highest peak of Chimborazo, where the barometer would have sunk below ten inches. The ocean seems to mark the boundary of that part of the atmosphere which is inhabited by winged insects, whose larvæ cannot find the necessary shelter and rest in its disturbed and unfathomable waters. But the depths of the ocean itself are not therefore untenanted; the waters everywhere teem with life, both vegetable and animal. Between the tropics the mariner, leaning over the side of his vessel, watches with interest the motions of the various species which every where swim around, and sees with pleasure the flying fish glancing through the air, where it is forced by its numerous pursuers to seek a momentary refuge. In the West-Indian seas, where the water is extremely clear, and its depth but small, he beholds on the bottom a thousand varieties of sea-worms, star-fish, snails and muscles, besides fish in prodigious numbers, and, as he sails along the surface of the water, he passes over whole groves of luxuriant fuci and other marine plants, together with immense masses of gorgonias, corallines, and alcyonias. He observes too with delight sponges, which, as large as shrubs, present the most beautiful play of colours to the eye, and, as they softly undulate with the motion of the waves, produce the pleasing illusion of his traversing fields covered with a profusion of flowers.

In other regions, notwithstanding the rapid course of his ship through the water, the navigator day after day beholds the ocean all around thickly covered with luxuriant sea-weeds, which, floating on the surface loose and unattached, grow without roots, and, to the inexperienced, convey the idea of rocks and shallows, dangers still far distant.*

Were we to seek an illustration of infinity in point of number, we would find it in those shoals of cods, herrings, pilchards, or sprats, whose moving and almost solid masses occupy many leagues. Even in the colder regions, and within the Arctic circle, the bosom of the ocean is scarcely less prolific of life. In some places, indeed, Captain Scoresby observed its waters tinged brown, as by a species of minute animalcula, which seeking light and heat near the surface, formed a living stratum not more than a few feet in thickness, but which covered a vast extent of the sea near the coast of Greenland. Captain Parry, while engaged in his bold attempt to reach the North Pole, observed that the little pools formed by the action of the sun on the surface of the snow and ice were full of animalcules.

On the coast of Greenland and other Arctic countries, the snow which covers mountains and valleys, and whose surface scarcely yields to the influence of the solar rays at midsummer, is in some places reddened* for miles together by a minute species of alga, which grows in vast quantity in the substance of the snow.

The infinity of animal life is exem-

* "The immense tract of sea, covered by floating fuci, has been spoken of by many authors, and especially by Humboldt, *Relation Historique du Voyage.*"

* "The redness of this snow, depending upon the presence of a minute alga, Protococcus nivalis, must be carefully distinguished from the red colour which Captain Parry observed on certain occasions, where the feet of his men or the bottoms of the sledges left impressions on the snow. This phenomenon was probably caused by a certain degree of transparency, which the surface-snow possessed in consequence of having been previously melted, and which allowed the transmission of the red rays alone through the parts forming the edges of the footsteps and sledge-tracks. The great obliquity of the sun's rays in so high a latitude favoured the production of this effect. No adequate explanation is given in the published account of Captain Parry's attempt to reach the North Pole over the ice. A most interesting paper, written by Count Xavier de Maistre, on the Colour of the Atmosphere and Deep Water, suggested this explanation. Vide Jameson's New Philosophical Journal, July, 1833."

plified in the most striking manner by a consideration of the *Infusoria,* an accurate and satisfactory account of which the student will find in a little work, illustrated by plates, lately published by Mr. Prichard, called the Natural History of Animalcules.

'The bare knowledge that there are myriads of atoms existing in a single drop of water, recreating and executing all their functions and evolutions with as much rapidity and apparent facility as if the range afforded to them was as boundless as the ocean, must carry with it an intensity of interest to the mind of every human being; of every one, at least, who is at all accustomed to meditate on the perfections of Nature, and to recognize and adore the hand which guides her through all the vast variety of her stupendous operations.' * * * * 'Until Dr. Ehrenberg adopted the expedient of introducing vegetable colouring matter into the fluid which supplies them with food, an experiment attended with the most successful results, these creatures were commonly supposed to be entirely devoid of internal organization, and to be nourished by the simple process of cuticular absorption. By the application of coloured substances, which, moreover, have been found to invigorate rather than to depress the animalcule, and to maintain it in the full exercise of all its functions, this erroneous notion is set at rest, and an internal structure has been discerned in some, equal to, if not surpassing, many of the larger intervertebrated animals, and comprising a muscular, a nervous, and, in all, probability, a vascular system, all wonderfully contrived for the performance of their respective offices.' "

Dr. Graves explores the vegetable world with equal comprehension, and the whole lecture is more than usually interesting.

---

### Death the Great Leveller.

The last few years have developed the most awful " SIGNS of the TIMES" without wars, or rumours of wars, we have had revolutions—and, as—

" Coming events cast their shadows before," we have had REVELATIONS, which betoken wonders still more wonderful than anything that has yet happened. If the shades of Tierney and Canning still hover round the purlieus of Palace Yard, how must they be astonished to see a WELLINGTON and a PEEL turn out professed—nay RADICAL REFORMERS? Could the bust of Baillie, in the College of Physicians, become animated, how would it stare, and smile—or perhaps grieve, on hearing the revolutions and changes which TIME is working in the new temple of Esculapius? But the wonder of wonders is yet to be declared—that Sir Henry Halford, the star of medical conservatism—the prop of privilege—the standard of prerogative—and the saviour of his " ORDER," should broach, and preach the most revolting doctrines of revolution—anarchy—and annihilation, before an immense assemblage of princes, ministers, senators, philosophers, physicians, &c. &c. &c. in the year of our Lord 1835!!

Such, however, is the fact. One of the most *vital* principles of our glorious constitution is—" *that the King never dies.*" Yet, in face of this immortal and gratifying maxim, Sir Henry Halford had the uncourtly audacity, and the democratic effrontery to declare that, from the post mortem examinations, and the actual evidence of his own senses, he was convinced that Kings do die—have died—and, worst of all, *may* die in future!! Who after this will say that courtiers are prone to flattery? Not only were KINGS reminded of their fate, by the illustrious orator; but the magnates of the earth were apprized that DEATH knocks even at their doors occasionally. Whatever our contemporaries may say to the contrary, we are of opinion that this oration was one of the most moral and useful that was ever delivered in Pall Mall East. TRUTH so rarely reaches the royal ear, or the ears of great men, that Sir Henry Halford deserves great credit for the intrepidity with which he delivered most unwelcome information to an aristocratic audience. Still, the event is pregnant with anticipations of the future. If kings and princes and ministers change their sentiments, fade in their persons, and shuffle off this mortal coil, it is possible that institutions, which are the offsprings of frail humanity, may also obey the same laws—may suffer decay, and ultimately perish—or change their names and nature. We are serious when we express our conviction that the paper of the president was well adapted to the majority of the audience. A dry medical disquisition would have been inappropriate to such an assemblage—and the *morale* of the address was of far more importance than the *physique*.

HENRY the EIGHTH of blessed and pious memory, was proved to be beautiful and manly, by the size of his arm-chair, the picture of Holbein, and the length of the coffin. Although he neither spared man in his anger nor woman in his lust, and was therefore an able auxiliary of that fell destroyer of all things—TIME, yet the old man and his scythe had no respect to the person of the handsome MONARCH. Though a dabbler in physic, he became corpulent and unwieldy—and notwithstanding that he composed a whole pharmacopœia for the benefit of his liege subjects, he got dropsical himself, and died, as history relates, with ulcerated legs, and in great sufferings! The orator humanely passed over the *frailties*—some fastidious historians would call them *crimes*—of the great monarch, and, on the principle—" *de mortuis nil nisi bonum*"—only portrayed his beneficent acts of *dispensation*, in the way of physic, to his subjects.

To his *prime minister*, WOLSEY, the amiable sovereign gave some excellent directions how to avoid the *sweatiug sickness*—an advice which may be necessary, however, unsuccessful, in our own times! But although Wolsey appears to have escaped the epidemic miasm that consigned so many thousands to the tomb, he was unable to resist the *moral* miasma, or *malaria* that issued from the mouth of his gracious master! Nay, it appears that no pestilential breath was necessary. A single glance from the eye of the sovereign gave every courtier his cue, and poor Wolsey found that, had he served his God as faithfully as he had served the vice-gerent of the Deity on earth, his death-bed would have been more happy! Sir Robert Peel, too, may have taken a good moral lesson from this discourse—but he has a better prospect than the Cardinal: he may serve his King and his country, without neglecting his God.

Wolsey's prediction of the time of his own death, and that, too, by dysentery, was a mere chance. No disease is of more uncertain duration than dysentery, and none where the prognosis, as to the period of termination, is more difficult.

In his reflections on the death of EDWARD the SIXTH, Sir Henry paid a tribute to the memory of this intelligent and amiable young monarch, whose mental powers bore no proportion to the weakness of his frame. Sir H. mentioned that he had met with many examples, where ill health had led young persons to great reflection and precocity of intellect—" compensating them for the brevity of their earthly existence!" We too, have seen examples of this kind, but have never found any difficulty in accounting for the phenomena on moral, physical, and physiological principles. Ill health deprives the young person of those exercises and amusements enjoyed by his associates, and he naturally takes to study as a substitute, and in which he finds pleasure and amusement. He therefore makes greater progress than others of his own age, who have health to take corporeal exercise and enjoy juvenile sports. But ill health confers no other advantage on the mind than what results from the above circumstances. Nay, it is often attended with great disadvantages—the mind not unfrequently participating in the maladies of the body, and being rendered incapable of much improvement. Thus any chronic disease of the stomach, the liver, or the head, will render its victim unable to compete with even the dullest person in the possession of health.

CROMWELL'S end shewed the ruling passion—enthusiasm, or perhaps an insane belief in supernatural intercourse—even in death. He put more confidence in his own fanaticism—or superstition than in the skill of the doctor—and was, of course, deceived.

King Charles the Second did not die for want of doctors. After the apoplectic seizure he was bled, cupped, purged and *vomited*. He died on the fourth day, though fourteen physicians signed his prescriptions—and although the "*spiritus cranii humani*" formed a component part of the draughts which the merry monarch was doomed to swallow! On examination of the head, "a copious effusion of lymph was found in the ventricles and at the base of the cranium." There is no mention of any sanguineous effusion or clot of blood—nor of any hemiplegia, which would have been the natural consequence of pressure on the opposite side of the brain. The case was evidently serous apoplexy, and the probability is that the fourteen physicians greatly hastened, if they did not occasion, the King's death. It is very rarely that we see any benefit to the patient from multiplicity of councillors. In general, these overgrown councils do much harm. The series of harassing questions that are put to the dying sufferer, only embitter his last moments; and where there are many doctors it is expected that many remedies will be tried, and the wretch's stomach is thus overpowered with physick, when Nature cries for repose to the incapacitated organs of digestion! Such are the fruits of consultations, when the patient is "*in extremis*." In *chronic*, and as they generally are, *incurable* diseases, the matter is very little mended. The patient flies from doctor to doctor, or has one consultation after another, each ending in some new remedy or plan that has not been tried before. The consequences are obvious. The disease is accelerated rather than retarded in its march, and confidence is lost, first in individuals, and ultimately in the whole profession.

In the details of the Duke of Gloucester's last illness and death, Sir Henry incidentally touched on a subject that has caused great error in the minds of philosophers, moralists, and divines. These people, and the world at large, wonder at the great difference, in respect to intellectual composure, exhibited by individuals on their death-beds. This difference depends almost entirely on the organ affected at the conclusion of life. "As the *brain* (says Sir H.) was not affected, his mind was left at liberty to indulge its natural propensity to look into futurity, and to anticipate the fatal issue of the struggle of the body with the disease." But it is not merely the organ affected, but the *way* in which it is affected, that makes the difference. In the present number of the Journal, our readers will have seen the remarkable case of Mr. J. of Portland-place, where the brain was disorganized on one side, yet where the intellects were clear to the last moment. The intellectual *functions* were not disturbed, because there was no fever nor inflammation. The same was seen, on a large scale, in the late epidemic cholera. Those who died in the collapse, retained their faculties till the last, while those who lived till re-action and fever supervened, had their mental faculties disturbed as in any other fever. It is also in meningeal inflammation that the intellectual powers are more particularly involved.

---

### Why are we Right-handed?

The following are the opinions of Sir Charles Bell on this subject. They are, perhaps, more ingenious than conclusive.

"In speaking of the arteries which go to the hand, it may be expected that we should touch on a subject, which has been formerly a good deal discussed, whether the properties of the right hand, in comparison with those of the left, depend on the course of the arteries to it. It is affirmed, that the trunk of the artery going to the right arm, passes off from the heart so as to admit the blood directly and more forcibly into the small vessels of the arm. This is assigning a cause which is unequal to the effect, and presenting, altogether, too confined a view of the subject: it is a participation in the common error of seeking in the mechanism the cause of phenomena which have a deeper source.

"For the conveniences of life, and to make us prompt and dexterous, it is pretty evident that there ought to be no hesitation which hand is to be used, or which foot is to be put forward; nor is there, in fact, any such indecision. Is this taught, or have we this readiness given to us by nature? It must be observed, at the same time, that there is a distinction in the whole right side of the body, and that the left side is not only the weaker, in regard to muscular strength, but also in its vital or constitutional properties. The development of the organs of action and motion is greatest upon the right side, as may at any time be ascertained by measurement, or the testimony of the tailor or shoemaker; certainly, this superiority may be said to result from the more frequent exertion of the right hand; but the peculiarity extends to the constitution also; and disease attacks the left extremities more frequently than the right.. In opera dancers, we may see that the most difficult feats are performed by the right foot. But their preparatory exercises better evince the natural weakness of the left limb, since these performers are made to give double practice to it, in order to avoid awkwardness in the public exhibition; for if these exercises be neglected, an ungraceful preference will be given to the right side. In walking behind a person, it is very seldom that we see an equalized motion of the body; and if we look to the left foot, we shall find that the tread is not so firm upon it, that the toe is not so much turned out as in the right, and that a greater push is made with it. From the peculiar form of woman, and the elasticity of her step resulting more from the motion of the ankle than of the haunches, the defect of the left foot, when it exists, is more apparent in her gait. No boy hops upon his left foot, unless he be left handed. The horseman put the left foot in the stirrup, and springs from the right. We think we may conclude, that every thing being adapted in the conveniences of life to the right hand as, for example, the direction of the worm of the screw or of the cutting end of the auger, is not arbitrary, but is related to a natural endowment of the body. He who is left handed is most sensible to the advantages of this adaptation, from the opening of the parlour door to the opening of a pen-knife. On the whole, the preference of the right hand is not the effect of habit, but is a natural provision, and is bestowed for a very obvious purpose: and the property does not depend on the peculiar distribution of the arteries of the arm—but the preference is given to the right foot, as well as to the right hand."

No one will fail to acknowledge the ingenuity of the foregoing observations; yet we apprehend that some people will not feel quite convinced by the arguments and illustrations. If the superiority of the right side depends inevitably on organization, we cannot conceive how left-handedness could ever obtain. If on the will of our Creator, the difficulty is increased. Are animals right-handed and right-footed? Are not children, from infancy, taught to use the right hand, and put the right foot foremost? It is very true that the two sides of the body are not quite equal in all respects. The right side boasts of a LIVER—but the left has a HEART—which is not inferior to its contemporary in many respects. Suppose Adam and Eve had some theoretical or superstitious predilection in favour of the right hand—and their children continued this predilection. Would not the right hand, in the course of generations, become stronger—originally from exercise, but ultimately from hereditary disposition? Scrofula must have arisen accidentally:—but see how it is transmitted from parent to progeny.

---

### ANATOMY-BILL.

It appears that, at a meeting of medical students in Edinburgh, held on Saturday, the 7th of February 1835, where nearly 700 individuals attended, a long discussion ensued on the difficulty experienced (in the private schools particularly of anatomy) in the northern metropolis, as to bodies for dissection. The 7th clause, especially, of the anatomy-bill, was blamed as the grand cause of the difficulties in question. This clause leaves it optional with the undertaker, or other person charged with the possession of a dead body, to give up the said body for money, for favour—or not to give it up at all, according to his pleasure or prejudice. The following petition to the Secretary of State, was therefore agreed to.

"That this meeting considers the present Anatomy bill defective, inasmuch as it places no restraint on parochial authorities regarding the disposal of their unclaimed dead, and leaves them quite at liberty either to dispose of them as may best serve their own interest, or bury them if they choose to do so; and suggests that it should be made imperative on all parochial authorities, superintendants of hospitals, infirmaries, charities, &c. to forward a notice to the Inspector of Anatomy, of their district, whenever a dead body lies in their possession, under such circumstances as are pointed out by the Act."

There was some discussion as to the propriety of soliciting Mr. Wakley to support the prayer of the petition. On a shew of hands, the great majority was in favour of the solicitation, and it was agreed to accordingly. Some gentleman at the meeting asserted that no difficulties were experienced in Dr. Monro's class, and that this meeting was almost entirely composed of the students of private schools. We regret to learn this state of things in the northern capital. It is said that up to the date of the meeting—when more than half the session had passed away, several hundred students had not had even an extremity for dissection! We fear there is little chance of redress at present. There is not much to be expected from *our* legislature at any time—and less now than ever. The political parties are now so nearly equipotent, that no ministry, whether Whig, Tory, or Radical can have any predominant weight in parliament to carry measures that may be, in the slightest degree, unpopular. A compulsory clause, such as is solicited in the foregoing address, would raise a cry against the minister that brought it in, and therefore we need hardly expect such an amendment to the Act at present.

---

### Hospital for Stone.

An institution for the reception of poor persons suffering from stone has existed in the metropolis now during nearly two years, under the care and at the sole expense of Mr. Costello, and the benefit it has been the means of conferring on numerous persons, has induced several gentlemen to adopt measures for rendering the establishment a permanent one on a larger scale.

The presence of stone in the bladder constitutes one of the most dreadful maladies with which humanity can be afflicted. The unceasing anguish of the disease, the terror which a prospect of the cutting operation inspires, and the danger of that operation when performed, are not surpassed (if equalled) by the incidents of any other affliction.

The extraction of the stone through a deep wound purposely effected in the process of lithotomy has until lately been the only remedy for the disease in its maturity; and although many improvements have been introduced both in the manual part of the operation and the instruments employed, yet the average mortality resulting from its performance, in a large number of cases, including patients of all ages, has been little less than one in four. Humanity, however, might enforce the propriety of concealing the average mortality of lithotomy, were it the sole resource against stone. But thanks to modern surgery, a milder and an incomparably more successful mode of cure has been discovered. Thanks to that distinguished disciple of surgery, Civiale, of Paris, lithotrity is fast assuming the laurels which lithotomy could never wear. The reduction of the stone to powder, or small fragments, by means of instruments, which are not larger than common sounds, enables the surgeon to proceed to the cure without bloodshed, with little pain, and almost without danger. Since M. Civiale performed his first operation, more than five hundred sufferers have been relieved by lithotrity in France and England, and as a proof of the high opinion entertained of that operation in France, it is only necessary to state, that five years ago an hospital was opened in Paris for its application to the poor. In 1829 Mr. Costello, who had till then been the partner of CIVIALE's labours in France, introduced lithotrity into England, and since that period he has not only considerably improved the process, but has taken unusual pains to make his professional brethren in Great Britain familiar with the operation, describing and performing it gratuitously before them in almost every large town.

But although by these exertions, seconded by the aid of many fellow practitioners, and among others by Mr. KING, in his late "Comparison between Lithotomy and Lithotrity," he has succeeded to a great extent in establishing in the minds of the profession the superiority of lithotrity, yet much remains to be done to render its adoption general, and to confine the painful operation of lithotomy to the few cases in which it presents the only resource. England is not the country in which the merits of such a process can long fail of obtaining for it its due rank among the discoveries of the age, if the opportunity be afforded to the public of advancing its utility. With this persuasion, several gentlemen have resolved to submit to the notice of the humane and benevolent, the infant institution which already exists for the cure of stone, in the hope of being enabled to extend its benefits to the full amount which they are capable of reaching, and thus at once afford extensive relief to calculous patients, and procure the means of materially facilitating the perfection of the operation itself, at the same time rendering to the profession every available opportunity of becoming familiarized with the details and execution of the lithotritic process.

In making this appeal for public support, they beg to submit the following facts:—The average number of cutting operations for stone annually amounts in the London Hospitals to 47, and the estimate for England and Wales, exclusive of the metropolitan institutions, is 64. The total is 111, which, in a population of twelve millions, gives one case for every hundred and eight thousand persons. From the 111, forty may be subtracted as cases occurring in children, who cannot at present be regarded as eligible subjects for lithotrity or lithotripsy, but in whom the cutting operation is still demanded. There thus remains an average of but 71 patients, (exclusive of those of Ireland and Scotland) who require an operation to be performed for stone at the public medical institutions of the country.

The number of beds which would be necessary in an hospital of lithotrity to receive all these cases, may be estimated as follows:—If one month be taken as the average duration of the treatment of each case, and the cases occur with uniformity of time, six beds would be sufficient for the purposes of the institution, and in twelve months the whole of the average number of patients could become inmates of the hospital. Supposing, however, this extent of accommodation to be doubled, it is doubtful if in the compass of any establishment so much good could be effected by such trifling means as those which would be sufficient to furnish and provide the proposed hospital.

It is also proposed, as soon as sufficient funds are obtained, to extend the objects of the institution to the treatment of some other diseases, which, like stone, require the concentrated care of the surgeon and nurse, and thus to assimilate it to some which exist on the continent, for the especial promotion of medical science, by recording the phases, subjugation or progress of maladies which cannot be so closely watched according to the usual modes of proceeding in large public hospitals. It is calculated that such an establishment in every sense of the word, of the highest value to the community, might be realized at a trifling expense, and it is confidently hoped from the known liberality of the people of this country that so beneficial an addition to our charitable institutions will meet with due support.

The poor will be received as heretofore by Mr. Costello, until such time as the house already hired for the purpose shall have undergone the necessary alterations.

DR. JAMES JOHNSON, *Physician.*
MR. COSTELLO and MR. KING } *Surgeons.*

